# Enablers and barriers associated with high MMR uptake among the Finnish Somali population

**DOI:** 10.1093/eurpub/ckac129.106

**Published:** 2022-10-25

**Authors:** I Hussein

**Affiliations:** Finnish Institute of Health and Welfare, Helsinki, Finland

## Abstract

**Introduction:**

In Finland, the Somali-speaking community is the fourth largest migrant group after Russian, Estonian, and Arabic-speaking communities. Finland is home to 21,000 Somali-speaking individuals, with around 70 % of them living in the capital region. Even though vaccine uptake in most ethnic minority communities is usually lower, MMR coverage in Finnish Somali children is partly higher than that of ethnic Finns.

**Methods:**

We interviewed 30 Somali-origin mothers living in the capital region of Finland in terms of childhood vaccination acceptance, and in specific, issues affecting their children's MMR vaccine uptake. All interviews were conducted by a Somali researcher either in Somali or Finnish language. The interviews were recorded, transcribed, translated, and analysed using thematic analysis of the transcripts. To understand the enablers presented by the health care system in Finland, 10-15 public health nurses working in maternal and child health clinics in the capital region will be interviewed.

**Results and discussion:**

Preliminary results show that trust in the health care system is the main enabler of high MMR vaccine coverage in the Somali population - even when mother's knowledge of the vaccine was very limited. In terms of information sources, mothers indicated that they ask, and trust maternal and child health clinics in all vaccine-related information- often more than any other source, including the internet.

**Conclusions:**

This study enhances our understanding of system level enablers in the Finnish Somali community regarding MMR vaccination. The results of this study are applicable to improve vaccine uptake in other underserved minority communities throughout Europe.

